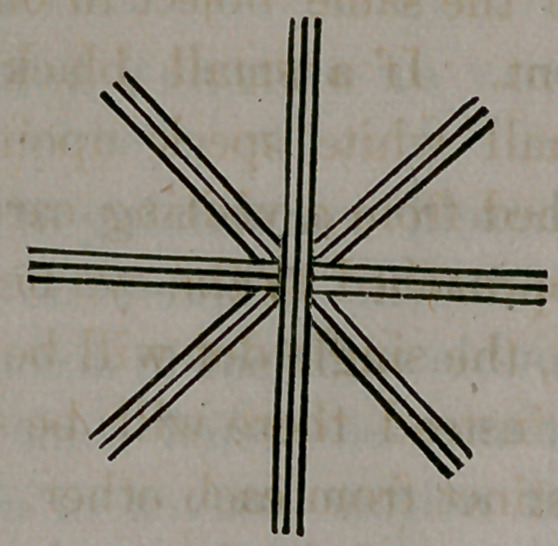# Astigmatism

**Published:** 1874-02

**Authors:** Swan M. Burnett

**Affiliations:** Knoxville, Tenn.


VOL. IV.—FEBRUARY, 1874.—NO. 2.

### Original Communications.

### ASTIGMATISM.

BY SWAN .M BURNETT, M.D., KNOXVILLE, TENN.

Sir Charles Bell once wrote a Bridgewater treatise upon the "human hand," which became famous. The object of these Bridgewater essays is to show in the various works of nature, the evidence of design, and to deduce from this fact, the necessity of a designer. Sir Charles did his duty well. It had occurred to very few, perhaps to look upon the hand as anything more than a very useful and convenient instrument with which to minister to our necessities, and it was only those who were deprived of its essential service that were fully aware of the very important and indispensable office it held in the human economy. In this essay however, Sir Charles Bell, showed that not only was the human hand a most useful and necessary member of the body, but by considering minutely and in detail, all its offices, and the accurate adaptation of the individual parts to the performance of their various functions, and the harmonious and economical combination of these individual functions, into a great whole, deduced the most convincing proof of design.

The existence of a designer was thus rendered necessary and a recognition of the fact made imperative.

No one has ever thought it necessary to write thus in regard to the human eye—for it has been, from time immemorial almost, looked upon, as the most perfect organ in the body, and as showing forth, more perhaps than any other, the great wisdom and goodness of the Creator.

And in truth such is the fact; but while we acknowledge this to be so, we feel compelled to say that we do not believe all the resources of the Creative Power were exhausted in the construction of the human machine; nor, in our opinion was it his purpose to make our organization a perfect one. Even to a finite intelligence many insufficiencies are apparent. The hand and arm, while performing many useful and necessary offices could, I think, without any detriment to their general usefulness, have been so fashioned as to relieve an irritation of the skin, between the scapulæ, thus obviating any necesssity for calling upon some friend to perform that office for us, or for resorting to the projecting corner of some friendly table, after the manner of the lower animals. Whether this faculty can be numbered among the "lost arts," we leave to the investigation of some disciple of Darwin, who may desire to "follow it thither with modesty enough and likelihood to lead to it." But who will be bold enough to make such assertions regarding the eye? Surely *it* is proof against any such charges of imperfection. But a proper regard for truth compels us to say that it is not. Like every thing in nature, as well as in art, it simply approaches to perfection, does not attain to it. Its degree is only relative, not positive. The human voice might have been made to compass more than an average of two octaves, and we might have been given the power to occularly perceive etherial undulations of a number below that which constitutes red, or above that which makes violet; and by adding more strings to corti's organ, our hearing power could have been made to exceed eleven octaves, which is about its present limit. The Creator had the power to give us this extension of faculty, but as it was not deemed essential to our well being, it was not granted us, and hence the only relative perfection of these and other organs of our system.

And especially is this true of the eye as an optical instrument.

It has been regarded until within a very recent period as the most perfect of optical instruments, and it was thought that man would never be able to construct an instrument approaching it in the accuracy of arrangement and completeness of its various parts. But as the physical laws governing the natural world were more thoroughly studied, and as the operation of these laws began to be applied to the elucidation of optical phenomena, various deficiences were discovered in the structure of the organ of vision, which quite exploded the idea of its perfection as an optical instrument. So many and so great are these imperfections that a celebrated German Scientist (Helmholtz) has said, "If an optician wanted to sell me an instrument which had all these defects, I should think myself quite justified in blaming his carelessness in the strongest terms, and giving him back his instrument."

This may sound startling to some, but the truth of the statement has been verified time and again, and by numerous individuals. The proof of some of them is within the reach of all. If we look through a small pin hole in a card, at a bright sky, numberless dark colored spots, granules and strings will dance before the eyes. These are due to opake particles in the vitreous fluid, or imperfections in the lens, which would certainly not be the case if the eye were perfect. If Mr. Tolles or Mr. Beck, or any of our microscope makers were to send us an objective made of imperfect glass, with specks of dirt or air bubbles in its substance, I think we would accuse him of excessive carelessness, and send him back his instrument as Helmholtz said he would do. A star, we know has not the rays which the optical image we get of it makes it appear to have. These rays are due, it has been demonstrated, to a faulty construction of the lens, to which we will refer furtheron.

Can man then construct an optical instrument more nearly perfect than the eye? Such is undoubtedly the fact, but that does not by any means prove the superiority of the work of man over that of nature, for the eye is something *more* than an optical instrument, it is likewise an organ of sense, and by virtue of being an organ of sense, is able to overcome many of its defects as an optical instrument; and to such an extent is this the case that, after some considerable education of the visual faculty, we are able to judge pretty accurately of things as they actually are. I would therefore advise any one who may have become dissatisfied from reading these statements, with his visual organs, to do nothing rash—for though he might exchange his eyes for an instrument constructed in more strict accordance with optical laws, he would fail most signally in getting one to answer all the purposes the eyes serve, imperfect as they are.

Some of these defects however, are occasionally of such high degree that we are not able unassisted by art, to overcome them, and then they amount to a serious inconvenience, and are classed among the diseases.

To one of these imperfections of the organ of vision, we desire to call attention in this article. It is called

*ASTIGMATISM,*

and being derived from two greek words—*a*, privative, and *stigma*, a point—means that rays of light emanating from a single point—called from this circumstance *homocentric*—are not united after their refraction by the eye in another point.

There are very few eyes that have not this imperfection to a greater or less extent, though it does not attain a degree such as to become troublesome, except in a small per-cent. of cases. Upon the whole, it does not fall under the observation of physician so frequently as either Myopia or Hyperopia. The following experiment will show what we mean by Astigmatism, better perhaps than a description, and will also show that it is present in many eyes that are considered healthy. In a square frame cross two fine wires or horse hairs at right angles to each other. Gradually remove the frame from the eye, and when it approaches to the farthest point of distinct vision, in most eyes it will be found that one of the wires will become indistinct before the other. In this we have discovered that the focus of the eye is not the same for horizontal and vertical lines. Astigmatism may then be defined to be—*that condition of the eye in which the refraction is not the same in all the meridians.*

*HISTORY.*

The first description of Astigmatism approaching at all to accuracy, we find made by Thomas Young, one of England's greatest Savants, and the originator likewise of the present accepted theory of accommodation. He describes it as it occurred in the person of himself, in the Philosophical Transactions for 1793.

His case was a peculiar one in two particulars. In the first place, in the experiment which we have just described, the horizonal wire was the first to disappear, whereas the contrary is commonly the case, and secondly, as he himself demonstrated, the error in refraction had its seat in the lens instead of the cornea, where it is found as a rule in this variety of astigmatism.

A more complete description is given by Airy, the Astronomer Royal of England, in the Trans: Camb: Phil: Soc: 1827. He had a compound Myopic Astigmatism, and so closely and accurately did he study his case, that he was enabled to calculate theoretically the form and strength of the glasses necessary to correct the refractive error, which upon application they were found to do.

Other cases after this were noted from time to time, but it was not until the subject was taken hold of by Donders and Helmholtz, that it received that thorough elucidation which renders it one of the most interesting and best understood chapters in ophthalmology. It is to these two and to Prof. Knapp, now of New York, that we are indebted for most of our recent knowledge of the subject. The natural division of astigmatism is into two

*FORMS*

which have been called *regular and irregular.* Of these, we will study the former first. It is called regular because the meridians of greatest and least refraction approach with greater or less regularity to the horizontal and vertical. The refractive error lies in this form, generally, in the curvature of the anterior surface of the cornea. To have refraction the same in all meridians, the cornea should be the section of a sphere; in regular astigmatism however, we find it to be the apex of an ellipsoid whose longest and shortest diameters are perpendicular or nearly so, to each other. The effect of such a refracting surface upon parallel or homocentric rays is evident. A bundle of parallel rays falling upon such a surface could, clearly, not be focussed at one point, but those passing through the meridian of shortest curvatue would unite and cross before those passing through the meridian of greatest curvature could be brought to a focus. The result of this will be circles of diffusion and a distortion of the image. Take as an example a piece of coin. If the refractive error, as is commonly the case, is such that parallel rays are focussed on the retina only in the horizontal meridian, when the object is placed at an infinite distance, or where the rays preceeding from it are parallel, the coin will not appear circular, but oval, and will be drawn out in the direction of the meridian of shortest curvature—namely, vertically. If the coin now be brought to such a distance that the vertical meridian will unite the rays upon the retina, the coin will be elongated in the opposite direction, viz: horizontally. In the former case the parallel rays united and crossed before they reached the retina and formed circles of diffusion in a vertical direction, while in the latter the divergent rays not having reached a focus formed diffusion circles in a horizontal direction. If the coin is viewed at a distance intermediate between these two, it will appear circular, but as neither of the meridians is in focus, the image will appear blurred in all directions, and its outlines will be indistinct. The same phenomena are apparent when a square object is used. It will appear as a figure oblong in the horizontal or vertical meridian according as it is viewed near at hand or at a distance. The same is true of the horizontal or vertical lines of letters. They are not seen with equal distinctions, and this constitutes one of the most frequent cases of complaint among astigmatics. Other phenomena are presented in the form of alteration in the character of the chromatic aberration of the eye—for it must be remembered that this is to numbered among the other imperfections from which the eye suffers. When a distant street lamp is looked at through a violet colored glass, if the eye is emmetropic and not astigmatic, the flame will appear red fringed with blue. The reason of this is, that the violet glass permitting the passage of only the blue and red rays, and the eye not being achromatic the blue rays are focussed and diverge again before reaching the retina, and form a border to the red, which being less refrangible are united on the retina, making a clear image. To an astigmatic eye the appearance is different. The flame will be bordered above and below by either red or blue, and on the sides by the opposing color, according as the refraction is greater in the horizontal or virtical meredian. The explanation of this, is that while the vertical meridian brings one color to a focus on the retina, the horizontal meridian on account of its different curvature will cause the rays passing through it to be so refracted that the retina will lie in that merian in the focus of the opposite color. The regular from of astigmatism may be modified in various ways, and it is necessary that we study the

*VARIETIES*

which it may present in order both for a clearer and more satisfactory method of studying it, as well as a more ready and certain determination of the glasses necessary for its relief. The modifications of regular astigmatism are three, as follows: One meridian may be emmetropic or normal, and the other either Myopic or Hyperopic; or one may be Myopic and the other Hyperopic; or there may be a difference of the same error of refraction in both meridians. Donders division is into

*FIRST—MYOPIC ASTIGMATISM,*

which has two subdivisions: *Simple Myopic* (Am.) where one meridian is E. and the other M., and *Comp: Myopic* (M and Am) in which there is a general Myopia, but greater in one meridian than in the other. Thus if there is in the vertical meridian $M=\frac{1}{5}$ and in the horizontal $M=\frac{1}{10}$—there will be a general Myopia of $\frac{1}{10}$ and a Myopic Astigmatism of $\frac{1}{10}$; written M $\frac{1}{10}$ and Am $\frac{1}{10}$.

*SECOND—HYPEROPIC ASTIGMATISM,*

which is as above, again subdivided into *simple*, where there is E in one meridian and H in the other (Ah) and *compound* where there is a difference of H in the two meridians. As an example let in the vertical meridian $H=\frac{1}{4}$, in the horizontal meridian $H=\frac{1}{6}$. Here the general $H=\frac{1}{4}$ and $Ah=\frac{1}{12}$. It is written as above H $\frac{1}{4}$+Ah $\frac{1}{12}$.

*THIRD—MIXED ASTIGMATISM*

of which there are two forms,—*a—with predominating M:* (Amh).

Example: In vertical meridian M=1/1[illegible], in horizontal meridian $H=\frac{1}{30}$, Amh would then be $\frac{1}{15}+\frac{1}{30}=\frac{1}{10}$. *b—with H predominant*

(Ahm). Example: vertical meridian $M=\frac{1}{12}$, horizontal meredian $H=\frac{1}{6}$, $Ahm=\frac{1}{12}+\frac{1}{6}=\frac{1}{4}$.

*The cause of Regular Astigmatism* is to be found, as a rule in the cornea, the lens not often playing any part in its production.

Regular Astigmatism, as may be inferred from the foregoing remarks, is, as a law, congenital. Astigmatism may be acquired it is true, but then it is with the rarest exceptions, of the irregular form. It is commonly but a part and parcel of a general arrest of development, and is nearly always, when of a high degree, associated with some other malformation, and especially with an anomalous development of the bones of the cranium. Even in the normal regular form—that is, that small degree which is common to most eyes—there is an associated form of the skull that is pretty constant. This form of the skull varies, according to Wecker, who has written an interesting communication on the subject, (Klinisch: Monats; fur Augenheild, June, 1870,) according as the greater curvature lies in the horizontal or vertical meridian. The form of the skull which characterizes the Teutonic race, has as its normal astigmatism, the shortest curvature in the vertical meridian. The researches of Javal and others show that when the normal astigmatism takes the other form, having the shortest corneal curvature in the horizontal meridian, there is associated with it a corresponding change in the shape of the skull. The kind of astigmatism, under which any nation or people labors, will of course influence their choice of written and printed characters. Those lines which lie in the direction of the faulty meridian, will be broader and more strongly marked than those in the normal meridian. Take the Roman letter as an example. Our faulty meridian is the vertical, and consequently we have our letters with their broadest strokes in the vertical direction. The Hebrew characters have the broad strokes in the horizontal direction, and from this it is argued that their normal astigmatism was the opposite of ours, with the anomalous refraction in the horizontal meridian, and that there must have been in them a corresponding difference in the formation of the skull. The law laid down by Wecker, is that "the meridian of shortest curvature corresponds with the diameter of the skull, which shows an anomalous shortening."

Its ethnological significance, if these can be established as facts, is patent. We have only to study the written or printed characters, and we know the astigmatism normal to that people, and thus indirectly the general shape of the skull.

Astigmatism is doubtless hereditary as well as congenital, though it is highly probable that incidental causes operating during the development of the eye exercise a large influence in its production.

### *DETERMINATION OF REGULAR ASTIGMATISM.*

A patient presents himself, complaining of an indistinctness of vision, existing since childhood. There is no evidence of present or past inflammatory trouble sufficient to account for the deterioration of vision, so we at once suspected some form of ametropia. Upon trial, we find that neither convex or concave glasses give complete relief, though they may give some; so it is clear that it is not a pure case of Myopia or Hyperopia. We may then strongly suspect astigmatism and pursue our examinations further as follows: We take a metallic disc with a slit in it about 1½ mm. broad, and holding it close before the eye, direct the patient to look through it at Snellens' test board. Revolving it about its own axis we cause the slit to come by turns before each meridian of the cornea. It will then soon be discovered that there is one meridian in which vision is best, and one in which it is worst. In regular astigmatism these approach to the vertical and horizontal, as has been mentioned before. Having found these meridians, we study each one separately in the following manner: Placing the slit in correspondence with the meridian of best vision, we try successively convex and concave glasses, to see if there can be any improvement, and if there is, the strength and character of the glass, giving this improvement, is to be noted. The slit is now placed before the meridian of worst vision and the character of its anomalous refraction and its degree is also to be noted. Then according to the formula given on page 69, the total astigmatism is easily calculated. This is the most natural, and upon the whole, the most accurate and satisfactory plan for the determination of astigmatism, particularly if we take care to obviate any interference of accommodation. In young persons whose accommodature is strong and active, it must be paralyzed by atropine as we do in determining Hyperopia. There are other modes, which though less accurate, are still sufficiently so in the majority of cases, for practical purposes and are also useful for the verification of other and previous examinations. One of these are to have the patient look at the accompanying diagram at a distance of ten or twelve feet.

The radii that are least distinguishable will mark pretty accurately the meridian or meridians that are faulty. If then, on making trial of cylindrical glasses, taking care to apply them with their refracting axes corresponding to the faulty meridians, we find one that corrects the anomaly, that glass will of course show the degree of astigmatism.

A very ready and handy method for determining the existence of astigmatism, has been brought forward by Dr. Knapp, of New York. It is the altered form of the optic disk as seen by the Ophthalmoscope. In examining by the direct method, giving the upright image, the disc in the meridian of greatest curvature appears more highly magnified, and in the meridian of least curvature least magnified, while the reverse is the case in the indirect method, with the inverted image. Hence, when the disc is viewed by both methods an elongation in opposite directions is noticed and the position of the least and most refracting meridians is readily determined.

*IRREGULAR ASTIGMATISM.*

This form of astigmatism is characterized by a difference of refraction in several meridians or in different parts of the same meridian. It, like regular astigmatism, is divided into *normal* and *abnormal*. Normal regular astigmatism we have seen, has its seat as a law, in the cornea; normal irregular astigmatism on the other hand, has its seat almost without exception in the lens. The abnormal form accompanies changes in the cornea, which are mostly acquired and the results of inflammatory affections.

The principal feature of *normal* irregular astigmatism is *polyopia urni-ocularis* or multiple images in one eye. That we can have more than one image of the same object in one eye is shown by a very simple experiment. If a small black spot upon a white ground, or better, a small white speck upon a black ground, (as the dust of lead scratched from a visiting card on a piece of black velvet) be gradually approached to the eye nearer than the nearest point of distinct vision, the single dot will be found by most persons to disappear, and instead there will be seen a small circle of faint dots, tolerably distinct from each other. This is the result of the anatomical construction of the lens, whereby the refraction of the different sections is made unequal, and as a consequence there is an image for each section. This is the case when the light is homocentric as when it proceeds from a star or a distant gas jet. The rays which these objects possess, and to which allusion was made at the beginning of this article, arise from this fault in construction. If the refraction was equal in all the sectors, the rays would not be present. And in addition to this, an astigmatism of each of the separate sectors has been experimentally demonstrated.

Normal irregular astigmatism however, seldom gives rise to any serious annoyance, because in the first place, objects are not as a rule viewed under circumstances necessary to its sensible production, and in the second place, because having the faculty of binocular vision, we are able, to a very large extent, to counteract in one eye the imperfections of the other.

In the *abnormal* form however, the case is different. Here the refractive error lies, as a rule, in the cornea, and is often of such an extent as to deprive the patient of the power of recognizing objects. Corneal irregular astigmatism is generally acquired, and as a rule inflammatory in its origin. Its most serious forms are *conical cornea and staphyloma.* Under such conditions, it is readily seen that of a bundle of parallel rays, hardly any two of them after refraction would be brought to the same point on the retina. In these cases, vision is essentially lost, for from the image, no correct or definite idea can be formed of the object. Of course the less the degree of conicity the less marked will be the symptoms, but even in slight cases the impairment to vision is very annoying.

The alteration in the corneal curvature, which is liable to follow cataract extraction, likewise produces an irregular astigmatism. The changes resulting from corneal ulceration, are also productive of the same result. In the case of resorption ulcers (ulcers with a transparent bottom) its influence upon vision is clearly marked. Here it cannot be opacity which interferes, for the ulcer is transparent, but the irregular astigmatism caused by it is so great sometimes as to interfere most seriously with vision.

The abnormal form may also have its seat in the lens. In this case it depends either upon a dislocation, or an unequal hardening of the different parts of its substance as we sometimes notice in incipient cataract.

### *TREATMENT OF ASTIGMATISM.*

In the varieties of the regular form, the treatment is by cylindrical glasses. These are the glasses to which we alluded under the head of "determination of astigmatism." They are so ground that their surface instead of being spherical, as in ordinary glasses, will be cylindrical; in other words, their surface instead of forming sigments of a circle, are sections of a cylinder. It is apparent that in a bundle of rays falling upon such a lens, those corresponding to the transverse diameter are refracted while these coinciding with the longitudinal axis are unaffected. The manner in which such a lens relieves regular astigmatisim is evident. It is best illustrated by examples. By the methods already detailed, we find, say, in the horizontal meridian Emmetropia; in the vertical $M=\frac{1}{10}$ (Am). What we desire now is a glass, which, while correcting the Myopia of the vertical meridian, will leave the refraction of the horizontal meridian unaffected. We accomplish this by applying a simple concave cylindrical glass—that is a glass plane on one side and concavely cylindrical on the other—with the concavity of ten inches focus before the eye, the axis of the cylinder being in the direction of the horizontal meridian. The refractive error is thus neutralized in the same manner as in the other forms of ametropia. In ordering a glass we write $-\frac{1}{10}$ c.

If the case is one of M+Am, and we find the total $M=\frac{1}{10}$ and the Am$=\frac{1}{10}$ for proper correction glasses will have to be spherical on one side of a strength sufficient to correct the general M., which in this case$=\frac{1}{10}$, and concavely cylindrical on the other in order to correct the additional M in the vertical meridian, which here is also $\frac{1}{10}$. We write then for a spherico-cylindrical glass thus, $\frac{1}{10}$s. ⊂ $\frac{1}{10}$c. If the astigmatism be mixed a bi-cylindrical lens will be necessary, that is convex on one side and concave on the other, with their axes of no refraction perpendicular to each other. In our case of Amh, where we had in the vertical meridian M$=\frac{1}{15}$; in the horizontal meridian H$=\frac{1}{30}$, we write for a glass $\frac{1}{30}$c⌐$\frac{1}{15}$e, and apply it with the concavity corresponding with vertical and the convexity with the horizontal meridian.

Where the glasses required are strong, allowance must be made for the distance of one-half inch, which the lens is placed in front of the eye. In positive glasses it must be subtracted from and in negative glasses added to the strength of the lens.

Where it is necessary to bring the far and near points to a certain distance, some alteration is of course necessary to be made in these calculations. We have not space here to enter into these, but sufficient has been given, we hope, to make clear the general principle for the adaptation of cylindrical glasses to the relief of regular astigmatism.

In irregular astigmatism it is not in our power to do very much for the patient in the way of glasses. Here operative procedures offer most for relief. In the case of conical cornea and staphyloma iridectomy or iridodesis will remove the pupil to a more evenly refracting portion of the cornea, and thus improve vision somewhat. The stenopaic apparatus, which allows only those rays to enter which lie in a certain direction, is also of much service. In the case of displacement or unequal hardening of the lens, it may be removed from behind the pupil by some of the operations for cataract.

## Figures and Tables

**Figure f1:**